# Sulfur-cycling diazotrophs dominate nitrogen fixation in seagrass sediments

**DOI:** 10.1128/aem.00993-26

**Published:** 2026-07-15

**Authors:** Lixia Deng, Zhicheng Ju, Jiawei Chen, Yuxuan Lin, Weiguo Zhou, Sangwook Scott Lee, Charmaine C. M. Yung, Hongbin Liu

**Affiliations:** 1 Department of Ocean Science, The Hong Kong University of Science and Technology 58207, Hong Kong, China; 2 Daya Bay Marine Biology Research Station, South China Sea Institute of Oceanology, Chinese Academy of Sciences, Shenzhen, China; University of Delaware, Lewes, Delaware, USA

**Keywords:** diazotrophs, sulfur-cycling bacteria, nitrogen fixation, seagrass sediment, metagenomics, metatranscriptomics

## Abstract

Diazotrophs, the microbes capable of fixing dinitrogen, are essential for providing bioavailable nitrogen that supports marine primary production. Traditionally, nitrogen fixation in seagrass sediments has been linked primarily to heterotrophic sulfate reduction, leaving the roles of other metabolic processes, like sulfur oxidation, largely unexplored. Here, we employed metagenomic and metatranscriptomic approaches to explore the distribution, metabolic capabilities, and activity of diazotrophs in sediments dominated by the seagrass *Halophila ovalis* in a subtropical bay in Hong Kong. Our results revealed significantly higher nitrogen fixation rates in seagrass-vegetated sediments compared to adjacent bare sediments, with peak rates occurring in subsurface layers, suggesting that seagrass sediments may serve as hotspots for nitrogen fixation. We recovered 305 metagenome-assembled genomes, including those of diazotrophic sulfur-cycling bacteria. Notably, sulfur-oxidizing *Gammaproteobacteria* and sulfate-reducing *Desulfobacterota* emerged as the dominant and active members of the diazotroph community in seagrass sediments, expressing specific genes related to both nitrogen and sulfur metabolic pathways. Furthermore, our findings suggest that sulfate-reducing *Desulfobacterota* likely drive the high nitrogen fixation rates observed in deeper sediment layers, while sulfur-oxidizing *Gammaproteobacteria* may play a crucial role in surface layers. This study underscores the important roles of both sulfate-reducing and sulfur-oxidizing bacteria in nitrogen fixation within seagrass sediments, revealing a complex interplay between nitrogen fixation and sulfur metabolism.

**IMPORTANCE:**

Seagrass meadows are vital blue carbon ecosystems found in coastal and estuarine regions, playing a crucial role in carbon sequestration and supporting marine diversity. Traditionally, biological nitrogen fixation, an essential process for supplying bioavailable nitrogen to living organisms, has been primarily associated with heterotrophic sulfate reduction in these ecosystems. Our research offers novel insights into the nitrogen-fixing microorganisms present in the sediments dominated by the seagrass *Halophila ovalis*. We found that both sulfur-oxidizing and sulfate-reducing bacteria contribute to nitrogen fixation processes in seagrass sediments. This study highlights the intricate connections between nitrogen and sulfur metabolic pathways, providing a more comprehensive understanding of nutrient cycling in coastal ecosystems.

## INTRODUCTION

Nitrogen (N) is the second most abundant element required for life on Earth (1). Despite the vast reservoir of N_2_ gas in the atmosphere, its bioavailability often limits the growth and productivity of terrestrial and aquatic ecosystems (1). Biological N_2_ fixation, a process carried out by specialized microorganisms known as diazotrophs, converts N_2_ gas into ammonia, thereby providing a critical source of bioavailable N for living organisms (1, 2). Marine diazotrophs are particularly important, accounting for nearly half of the global fixed N demand and regulating marine primary productivity (3, 4).

Extensive research has explored biological N_2_ fixation within seagrass meadows, which are vital ecosystems found in coastal and estuarine regions (5–7). These meadows support diverse marine life, enhance water quality, reduce disease risks, and serve as important blue carbon (C) sinks, thereby playing a crucial role in climate regulation (8–11). N_2_ fixation can occur in various parts of seagrasses, including leaves (12), rhizomes (7), and roots (6), as well as in the surrounding sediments (13). This process is energetically costly, consuming 16 ATP molecules for each N_2_ reduced (14). In organic-rich seagrass meadows, N_2_ fixation is closely associated with organic matter degradation, primarily through sulfate reduction (15–18). For instance, in the rhizosphere of seagrasses such as *Zostera noltii* and *Z. marina*, N_2_ fixation rates are influenced by organic C levels and can be significantly reduced by inhibiting sulfate-reducing bacteria (15, 17). Additionally, the connection between N_2_ fixation and heterotrophic sulfate reduction has been documented in the roots and rhizomes of *Z. noltii* and *Posidonia oceanica* (18, 19).

Seagrass sediments are generally recognized as organic-rich environments, where high N_2_ fixation rates have been observed (13, 20). Certain heterotrophic sulfate-reducing deltaproteobacterial taxa, such as *Desulfobulbus*, have been identified as the dominant diazotrophs based on the analyses of the *nifH* gene amplicons (21, 22). Moreover, the significant presence of sulfur-oxidizing bacteria has been noted in seagrass sediments, as indicated by 16S rRNA gene amplicons (23). This prevalence of sulfur-oxidizing bacteria, combined with the abundance of reduced inorganic compounds like sulfide generated during organic matter degradation, suggests a possibility that sulfur (S) oxidation could also serve as an energy source for the N_2_ fixation process in seagrass sediment ecosystems. Recent research has advanced our understanding further by recovering metagenome-assembled genomes (MAGs) from *Z. japonica*-colonized and unvegetated sediments (24). Notably, two of these genomes encode genes for both N_2_ fixation and thiosulfate oxidation, though their distribution, functional roles, and gene expression remain largely uncharacterized, reinforcing the potential coupling of S oxidation and N_2_ fixation in seagrass sediments.

Our current understanding of diazotroph communities in seagrass sediments primarily relies on *nifH* gene amplicon sequencing. While this approach has provided valuable taxonomic insights, it offers limited information on the functional activities of these microorganisms. In particular, the metabolic potential and activity of diazotrophic sulfur-oxidizing bacteria in seagrass sediments remain poorly understood, leaving the relationship between N_2_ fixation and S oxidation unclear. Furthermore, while the connection between N_2_ fixation and sulfate reduction is well-documented in seagrass ecosystems, the specific metabolically active sulfate reducers involved and their metabolic potential have yet to be explored from a genome-centric perspective in seagrass sediments.

In this study, we aimed to elucidate the roles of diazotrophic sulfur-cycling bacteria, including both sulfur-oxidizing and sulfate-reducing bacteria, in seagrass sediments through a comprehensive approach. We first characterized the physicochemical properties of the sediments and measured N_2_ fixation rates. Additionally, we employed metagenomic and metatranscriptomic techniques to explore the diazotroph communities present. We also assessed the metabolic potentials of diazotrophic sulfur-cycling bacteria by analyzing their MAGs. By integrating biogeochemical measurements with a multi-omics approach, this study provides valuable insights into the diazotroph communities in seagrass sediments, highlighting the important contributions of sulfur-cycling bacteria to N_2_ fixation within these vital ecosystems.

## MATERIALS AND METHODS

### Site description and sampling

The sampling site is situated in a *Halophila ovalis* seagrass meadow within the intertidal zone near San Tau Pier in Tong Chung Bay, Lantau Island, Hong Kong (22°17′14″ N, 113°55′31″ E) (Fig. S1). In August 2024, three replicate sediment cores were collected from both vegetated and adjacent unvegetated regions using a PVC sampling column after ebb (~16:00). After carefully removing surface seagrass plants, the sediment cores were sliced into four depth intervals of 3 cm each: 0–3, 3–6, 6–9, and 9–12 cm, resulting in a total of 24 samples. Subsamples for physicochemical properties analysis and N_2_ fixation rate measurements were placed in a portable cooler at 4°C and transported to the laboratory within 4 h for immediate analysis. Likewise, subsamples for DNA extraction were kept in the cooler at 4°C during transport and subsequently kept at −80°C until analysis. For RNA extraction, subsamples were flash-frozen in liquid N_2_, transported to the laboratory within 4 h, and stored at −80°C for further analysis.

### Physicochemical property analysis

Porewaters were extracted from fresh sediments using Rhizon samplers with a 0.1 μm pore size (25). The concentrations of sulfate (SO_4_^2−^) in the porewater were measured using ion chromatography (Thermo Fisher AQ-1100). Ammonium (NH_4_^+^), nitrate (NO_3_^−^), and nitrite (NO_2_^−^) concentrations were determined colorimetrically with a SEAL AA500 Nutrient AutoAnalyzer (Seal Analytical, Germany) following the manufacturer’s instructions. To analyze total organic C (TOC) and total N (TN) in sediment samples, the sediments were dried at 65°C to a constant weight, digested with 5% hydrochloric acid, and subsequently analyzed using an elemental analyzer (Eurovector EA3028) (26, 27). Physicochemical measurements were performed on pooled samples without replication; therefore, statistical comparisons were not performed, and observed differences should be interpreted as trends.

### Measurement of nitrogen fixation rates

N_2_ fixation rates were determined using the acetylene reduction assay (28). Approximately 50 g of fresh sediment was placed in a 125 mL polycarbonate bottle, which was then sealed with a septum closure cap (Thermo Fisher Scientific, MA, USA). To create an anaerobic environment, the bottles were flushed with N_2_ gas for 10 min. Subsequently, 5 mL of the headspace was replaced with acetylene (Air Products and Chemicals, Inc., China) before the bottles were incubated in the dark at 25°C for 24 h. After incubation, ethylene concentrations were measured by injecting 80 μL of headspace gas into a gas chromatograph (HP7890B, Agilent, USA) equipped with a flame ionization detector. The rates of ethylene production (acetylene reduction) were then converted to N_2_ fixation using a theoretical ratio of 3:1 (29) and normalized to the mass of sediment.

### Nucleic acid extraction and sequencing

Total DNA and RNA were extracted from sediment samples using the DNeasy PowerSoil Kit (Qiagen, Hilden, Germany) and the RNeasy PowerSoil Total RNA Kit (Qiagen, Hilden, Germany), respectively, following the manufacturer’s protocols. The quality of extracted DNA was assessed using the Qubit dsDNA Assay Kit with a Qubit 2.0 Fluorometer (Thermo Fisher Scientific). RNA quality and integrity were evaluated using a NanoDrop spectrophotometer (Thermo Fisher Scientific) and the RNA Nano 6000 assay kit in conjunction with the Agilent Bioanalyzer 2100 system (Agilent Technologies, CA, USA), respectively.

The qualified DNA and RNA samples were subsequently prepared for metagenomic and metatranscriptomic sequencing on the NovaSeq 6000 system (Illumina, San Diego, CA, USA), generating 150 bp paired-end reads (~15 Gb of raw data per sample). After sequencing, Trimmomatic v0.39 (30) was utilized to trim the metagenomic and metatranscriptomic data.

### Profiling the diazotroph community

Clean reads of all metagenomic samples were co-assembled using MEGAHIT v1.2.9 (parameters: --k-min 27 --k-max 147 --k-step 12) (31). The coding sequences of the resulting metagenomic assembly were predicted using Prodigal v2.6.3 (parameter: -p meta) (32). These coding sequences were then annotated using Diamond v2.1.6 (parameters: --sensitive -k 1 -e 1e-100 --id 50 --query-cover 75 --subject-cover 75) (33) against *nifH* sequences obtained from the Kyoto Encyclopedia of Genes and Genomes (KEGG) database (34). The *nifH* marker gene, encoding the iron protein subunit of nitrogenase, is extensively used to explore the diversity and distribution of diazotrophs across various environments (35, 36).

Sequences annotated as *nifH* genes were extracted and clustered at 95% sequence identity using CD-HIT v4.8.1 (37) to eliminate redundancy, thereby creating a non-redundant *nifH* gene catalog. The coverage of *nifH* genes in each metagenomic sample was calculated using CoverM v0.6.1 in “contig” mode (https://github.com/wwood/CoverM) (parameters: -min-read-percent-identity 95 -min-read-aligned-percent 75 -methods reads_per_base) based on the *nifH* gene catalog. Furthermore, the reads per base value of each *nifH* sequence was converted into transcripts per kilobase million (TPM) to represent the diazotroph community.

### Metagenomic binning

Clean metagenomic reads were aligned to their assembly using Bowtie2 v2.4.4 (default parameters) (38) to determine contig coverage. The assembled sequences were binned using MetaBAT2 v2.12.1 (39) and CONCOCT v1.1.0 (40), applying a contig length cut-off of 1.5 kb. The resulting bin sets were refined with the metaWRAP Bin_refinement module (parameters: -c 50) (41). All generated bins were combined and dereplicated to create a non-redundant set of MAGs using dRep v3.2.2 (parameters: -comp 50 -con 10 --P_ani 0.9 --S_ani 0.95) (42) at 95% average nucleotide identities. The completeness and contamination of the MAGs were assessed using CheckM v1.1.2 (43). Recovered MAGs with a completeness less than 50% and contamination greater than 10% were considered low quality and discarded. The chimerism of the MAGs was assessed using GUNC v1.1.0 (default parameters) (44). Taxonomic information for the MAGs was obtained using the Taxonomy Database Toolkit (GTDB-Tk) v1.6.0 (45).

### Phylogenetic analyses and functional annotation of MAGs

Maximum-likelihood trees for the MAGs were built using GTDB-Tk v1.6.0, based on the multiple sequence alignment of 120 bacterial and 122 archaeal marker proteins (46). Briefly, Prodigal v2.6.3 (parameter: -p meta) predicted amino acid sequences for the MAGs, which were then aligned to Pfam and TIGRfam hidden Markov models using HMMER v3.3 (http://hmmer.org/). ProtTest v3.4.2 (47) was employed to determine the optimal phylogenetic model, and a phylogenetic tree was constructed using IQ-TREE v1.6.12 (48) with the ultrafast bootstrap parameter “-bb 1000” (49). The resulting trees were visualized using the Interactive Tree of Life (iTOL; v6) (50).

The coding sequences from all MAGs were predicted using Prodigal v2.6.3 (parameter: -p meta) and annotated using Diamond v2.1.6 (parameters: --sensitive -k 1 -e 1e-100 --id 50 --query-cover 75 --subject-cover 75) against the KEGG database.

As the phylogeny of the *dsrAB* gene allows for the differentiation between oxidative and reductive *dsrAB* types, we mapped all genes retrieved from MAGs to a reference database (51) with Clustal Omega v1.2.4 (52) and constructed a maximum-likelihood phylogenetic tree using IQ-TREE v1.6.12 (48). Phylogenetic placement within the tree was used to classify the *dsrAB* gene into oxidative and reductive types.

### Identification of diazotroph MAGs

The identification of diazotroph MAGs was conducted using the Diaiden pipeline (https://github.com/jchenek/Diaiden) (53). Briefly, the coding sequences of genomes were predicted using Prodigal v2.6.3 (parameter: -p meta). These coding sequences were then annotated using Diamond v2.1.6 (parameters: --sensitive -k 1 -e 1e-100 --id 50 --query-cover 75 --subject-cover 75) against *nifH* sequences obtained from the KEGG database. Genomes with the *nifH* gene detected were classified as potential diazotroph genomes. The truncated average sequencing depth at 80% (TAD80) for each MAG was determined using CoverM v0.6.1 (-p bwa-mem --min-read-aligned-length 100 --min-read-percent-identity 95 --min-read-aligned-percent 70 --exclude-supplementary -m trimmed_mean --trim-min 10 --trim-max 90) (54). The relative abundance of each diazotroph MAG was calculated by dividing TAD80 by genome equivalents (GEQ), which accounts for variations in sequencing effort, MAG length, and microbial genome size across metagenomes (55). GEQ values were obtained from MicrobeSenses v1.1.0 (55).

### Metatranscriptomic analysis

Gene expression levels were determined by normalizing read counts by gene length and the median abundance of 10 universal single-copy phylogenetic marker genes of the prokaryotic community (K01409, K01869, K01873, K01875, K01883, K01887, K01889, K03106, K03110, and K06942) (56). Clean metatranscriptomic reads were aligned to the functionally annotated coding sequences from all MAGs using BOWTIE 2 v.2.4.1 with default settings. The resulting files were transformed into BAM format, after which CoverM v0.6.1 was utilized to remove alignments with less than 95% identity and 75% alignment coverage. Coverage profiles were subsequently generated using the filtered BAM files. The overall expression level for each MAG was calculated by summing the normalized read counts for all genes within its genome (57).

### Analysis of publicly available metagenomic data sets for seagrass sediments

Metagenomic data sets were compiled from 39 seagrass sediment samples collected across seven geographically diverse sites worldwide (Data S1 and references therein). Trimmomatic v0.39 (30) was utilized to trim the metagenomic data. Metagenomic binning, along with the identification and functional annotation of diazotroph MAGs, was performed as described earlier.

## RESULTS AND DISCUSSION

### Depth profile of physicochemical characteristics and nitrogen fixation rates in sediments

We conducted a detailed analysis to assess the vertical distributions of TOC, TN, NH_4_^+^, NO_*x*_ (NO_2_^−^ + NO_3_^−^), and SO_4_^2−^ in seagrass-vegetated sediments and adjacent unvegetated sediments at depths ranging from 0 to 12 cm in Tong Chung Bay during August 2024 (Fig. S2 and Data S2). Our results suggested that TOC, TN, and the C:N ratio in vegetated sediments (0.21–0.38%, 0.025–0.044%, and 9.3–10.4, respectively) tended to be higher at each depth compared to their corresponding depths in unvegetated sediments (0.15–0.25%, 0.021–0.03%, and 8.6–9.6, respectively), with peaks observed in the 3–6 cm layer for both sediment types (Fig. S2A through C). NH_4_^+^ concentrations were also higher across all depths in vegetated sediments (51.7–94.6 μmol L^−1^) than in unvegetated ones (20.4–36.9 μmol L^−1^), although they decreased with depth in both environments (Fig. S2D). The concentrations of NO_*x*_ (NO_2_^−^ + NO_3_^−^) were greater in the upper 0–6 cm layer of vegetated sediments (4.6–10.5 μmol L^−1^) compared to unvegetated sediments (3.1–4.7 μmol L^−1^), again peaking in the 3–6 cm layer in vegetated sediments (Fig. S2E). In contrast, SO_4_^2−^ concentrations were lower in the 3–9 cm layer of vegetated sediments (13.2–13.4 mmol L^−1^) compared to other depths (15.4–15.9 mmol L^−1^), while the same subsurface layer of unvegetated sediments exhibited relatively higher SO_4_^2−^ levels (15.4–15.5 mmol L^−1^) (Fig. S2F). Together, our results show that the studied seagrass-vegetated sediments exhibit elevated organic C content, a high C:N ratio, increased NH_4_^+^ levels, and reduced SO_4_^2−^ concentrations in the subsurface layers.

In terms of N_2_ fixation, rates were significantly higher in vegetated sediments, averaging 0.21 nmol/(g × d), compared to only 0.09 nmol/(g × d) in adjacent unvegetated sediments (*P* < 0.05, by pairwise Mann-Whitney tests, Fig. S3). Notably, N_2_ fixation rates were undetectable in the surrounding water. Previous studies have similarly indicated high N_2_ fixation activity in sediments vegetated by seagrasses compared to bare sediments (17, 58), suggesting that seagrass-vegetated sediments serve as hotspots for N_2_ fixation. However, the N_2_ fixation rates measured in our study using the acetylene reduction assay should be interpreted with caution and may have been underestimated (13). While this method is widely used in various ecosystems due to its logistical simplicity and cost-effectiveness, concerns have been raised (59). Specifically, the presence of acetylene may inhibit other microbial processes in sediments, including denitrification and sulfate reduction, and potentially even N_2_ fixation itself (60–63). Future studies should integrate the ^15^N_2_ tracer technique to provide a more accurate and comprehensive assessment of N_2_ fixation processes.

**Fig 1:**
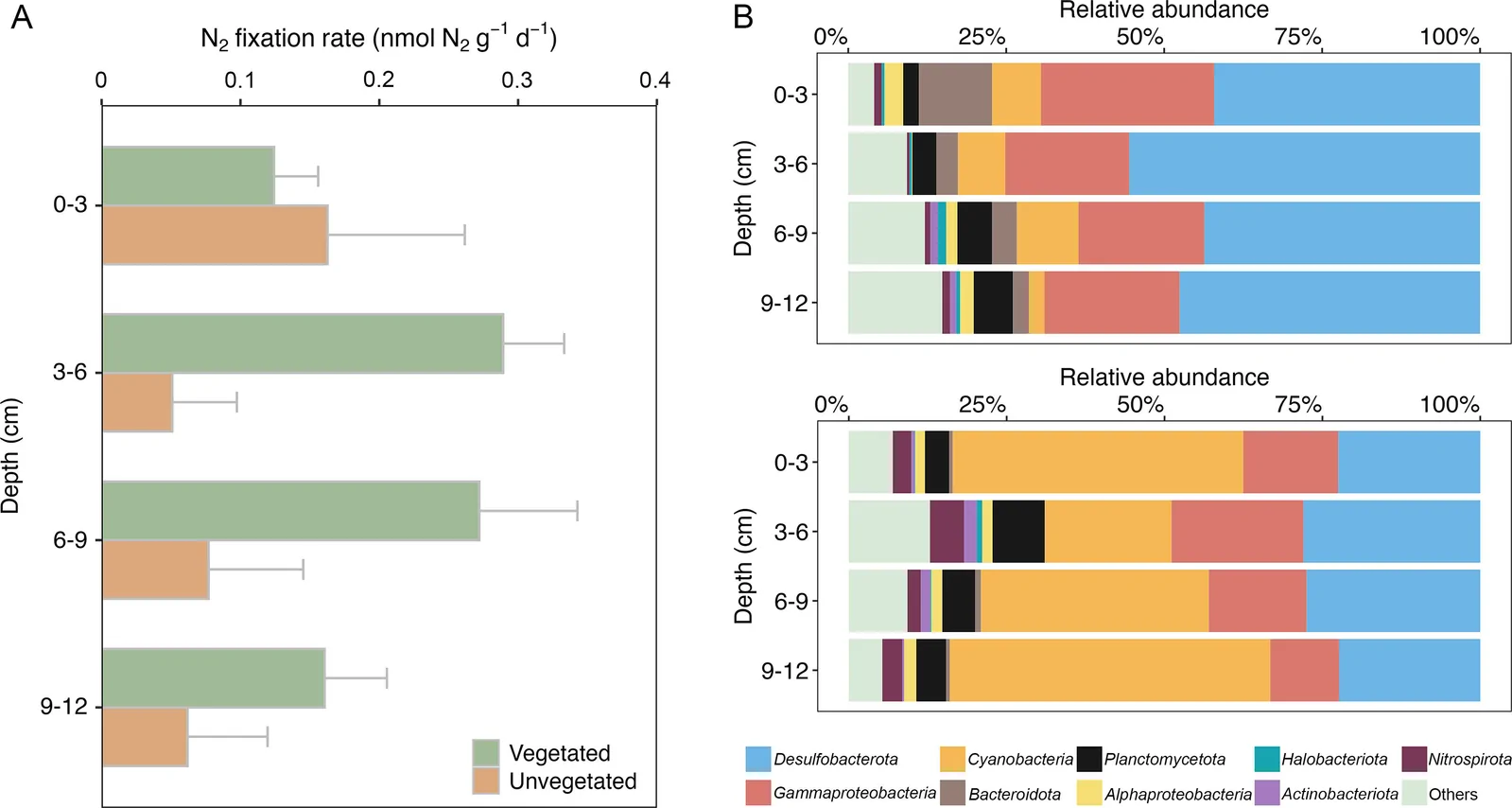
Vertical profiles of nitrogen fixation rates and diazotroph communities in seagrass-vegetated and unvegetated sediments. (**A**) Nitrogen fixation rates across different depths, shown as acetylene reduction (nmol N_2_ g^−1^ d^−1^). Values are represented as mean ± standard deviation of three biological replicates. (**B**) Relative abundances of *nifH* genes associated with different taxonomic groups in seagrass-vegetated sediments (top) and unvegetated sediments (bottom).

We also observed a depth-dependent variability in N_2_ fixation rates in vegetated sediments (*P* < 0.05, one-way ANOVA, Fig. 1A). In these sediments, rates fluctuated between 0.10 and 0.35 nmol/(g × d), peaking in the 3–9 cm layer, although significant differences were only observed between 0–3 and 3–6 cm layers, as well as between 0–3 and 6–9 cm layers (*P*<0.05, Fig. 1A). In contrast, N_2_ fixation rates in unvegetated sediments ranged from 0 to 0.27 nmol/(g × d), with highest average rates occurring in the uppermost 0–3 cm layer. However, no significant differences were observed among various depths in unvegetated sediments (*P* >0.05, Fig. 1A). This depth-dependent variation in N_2_ fixation in vegetated sediments was similarly reported in sediments colonized by the seagrass *Z. marina* (17), where the highest rates were also found in subsurface layers.

### Depth profile of diazotroph communities in sediments

To gain insights into the diazotroph communities, we mapped the metagenomes against the non-redundant catalog of *nifH* genes (Data S3). In vegetated sediments, the predominant diazotrophic groups were *Gammaproteobacteria* and *Desulfobacterota*, which together accounted for over 60% of the total diazotrophs across different sediment depths (Fig. 1B). Specifically, they made up 70% in the 0–3 cm layer, 75% in the 3–6 cm layer, 64% in the 6–9 cm layer, and 69% in the 9–12 cm layer. Within these communities, *Desulfobacterota* comprised 42–55% of the total diazotrophs, while *Gammaproteobacteria* represented 20–27%. Our findings aligned with previous studies that utilized *nifH* gene amplicon sequencing to explore diazotroph communities. For instance, Sun et al. (21) reported that in the vegetated sediments of the seagrass *Z. marina*, *nifH* phylotypes related to *Desulfobacterota* (*Desulfobulbaceae*) and *Gammaproteobacteria* (*Geobacteraceae*) were dominant. Similarly, Zhou et al. (22) found that *Gammaproteobacteria* and *Desulfobacterota* were the prominent groups among both total and active diazotroph communities in the vegetated sediments of *H. ovalis*. These results suggest that *Gammaproteobacteria* and *Desulfobacterota* are likely the primary N_2_-fixing groups in seagrass sediments. In contrast, unvegetated sediments exhibited a different community composition, where *Cyanobacteria* emerged as the most prevalent group, constituting 20–51% of the total diazotrophs at various sediment depths (Fig. 1B). *Desulfobacterota* also contributed a significant proportion in these sediments, accounting for 22–28% of the total diazotrophs across different layers (Fig. 1B).

### Genome-centric multi-omics approach uncovers the role of diazotrophic sulfur-cycling bacteria in seagrass sediments

**Fig 2:**
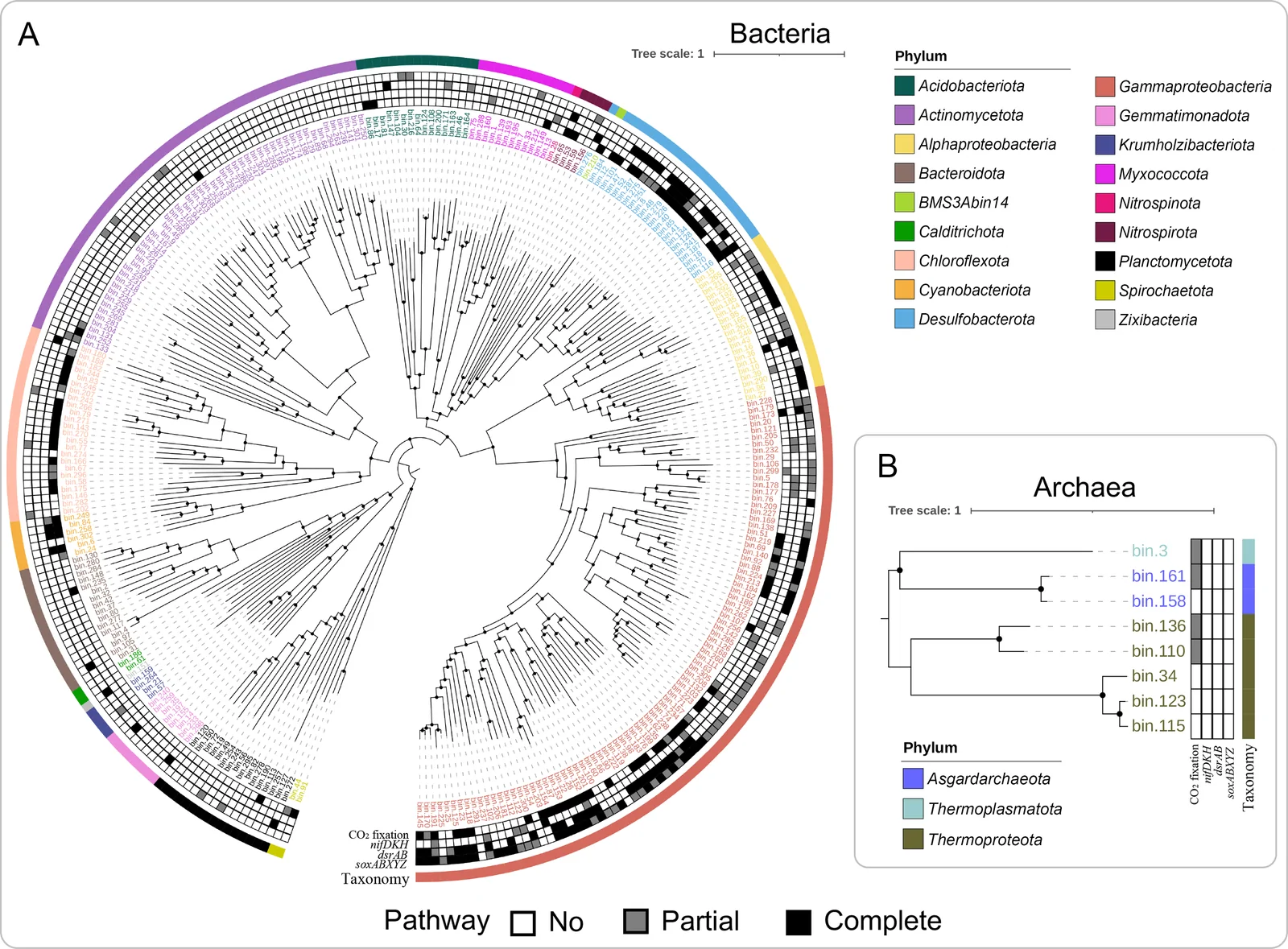
Phylogenetic reconstruction of 305 dereplicated MAGs derived from seagrass-vegetated and unvegetated sediments. Phylogenomic analysis of (**A**) 297 bacterial and (**B**) 8 archaeal MAGs. The inner concentric rings/bars indicate the presence/absence of genes related to carbon fixation, nitrogen fixation (*nifHDK*), dissimilatory sulfur metabolism (*dsrAB*), and thiosulfate oxidation (*soxABXYZ*). The outer ring/bar represents the phylogenetic classification of the MAGs at phylum level, with color representing different phyla. Black circles at the nodes show bootstrap values greater than 70%, and scale bars represent the average number of substitutions per site.

We recovered 305 non-redundant population genomes using metagenomic assembly and binning techniques, each exhibiting over 50% completeness and less than 10% contamination (Fig. 2 and Data S4). These genomes were classified into 21 phyla according to the Genome Taxonomy Database (GTDB), with nearly half affiliated with the phyla *Desulfobacterota* and *Gammaproteobacteria*. To assess taxonomic novelty, we utilized GTDB-Tk v.2.3.2 alongside the reference database R10-RS226 (64). Among the recovered MAGs, we identified 8 from undescribed families, 80 from undescribed genera, 213 from undescribed species, and only 4 from previously characterized species.

To evaluate the genetic potential of these MAGs for crucial biogeochemical functions within seagrass ecosystems, we annotated their open reading frames using the KEGG database, focusing on genes involved in the cycling of C, N, and S (Fig. 2 and Data S5). Given that the dissimilatory sulfite reductase enzyme (DsrAB) can mediate either sulfite reduction or sulfide oxidation, we conducted a phylogenetic analysis to determine the *dsrAB* type encoded in our MAGs (56). Our analysis revealed that the *dsrAB* genes from *Alphaproteobacteria* and *Gammaproteobacteria* were classified as oxidative types, while those from other MAGs, including *Desulfobacterota*, were grouped under reductive types (Fig. S4). A significant proportion of binned *Gammaproteobacteria* presented genes associated with N_2_ fixation, CO_2_ fixation via RuBisCO, dissimilatory S metabolism, and thiosulfate oxidation mediated by the *soxABXYZ* genes (Fig. 2). Most MAGs from the *Desulfobacterota* phylum contained genes for N_2_ fixation, CO_2_ fixation through the Wood–Ljungdahl pathway, and dissimilatory S metabolism (Fig. 2).

**Fig 3:**
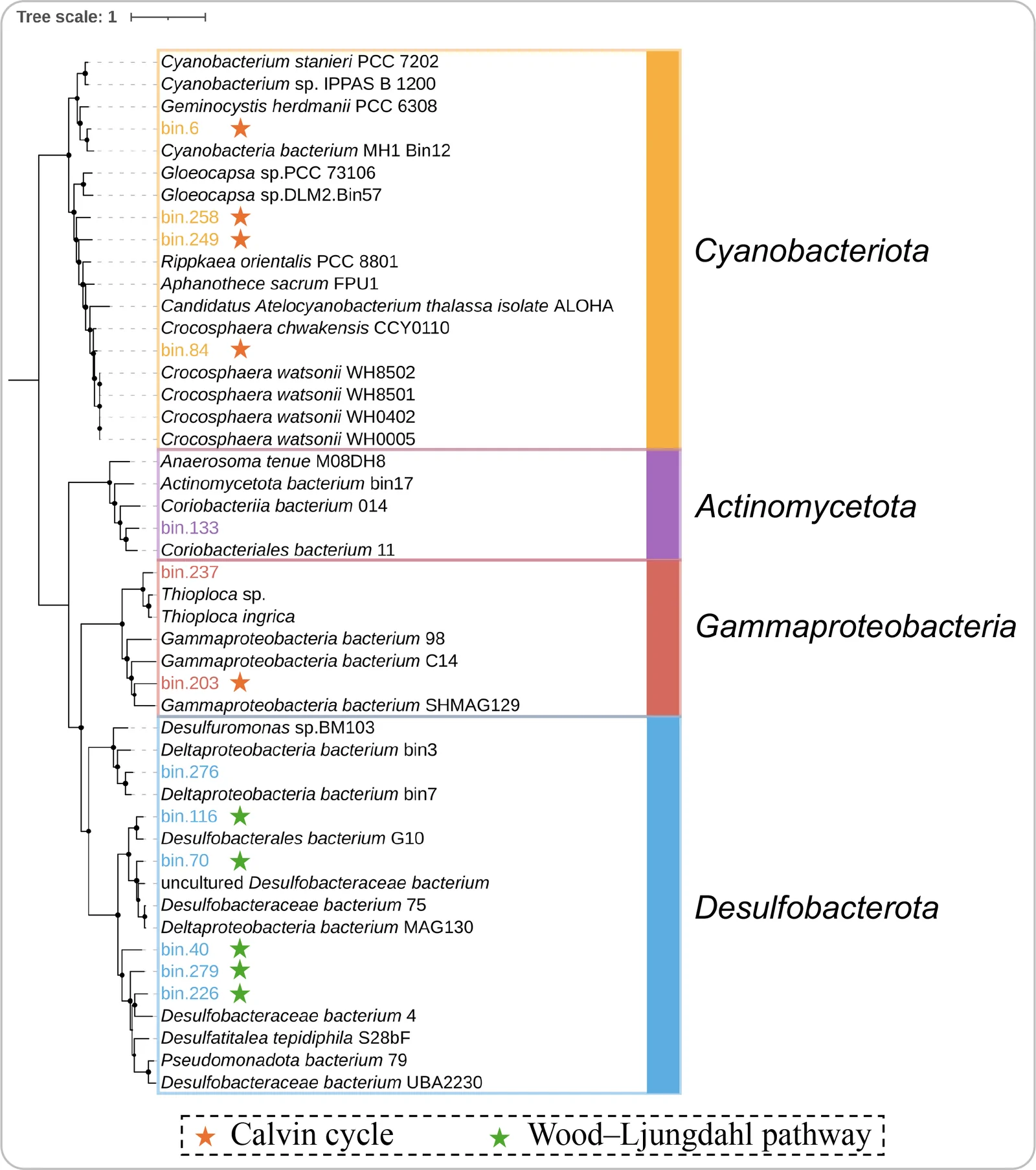
Maximum-likelihood phylogenetic tree of 13 diazotroph MAGs. MAGs are color-coded based on their *nifH* homolog groups at the phylum level. Different colored stars represent different carbon fixation pathways. Black circles at the nodes show bootstrap values greater than 70%, and scale bars represent the average number of substitutions per site.

Among the recovered genomes, we identified 13 MAGs across 4 phyla that contain the *nifH* gene, indicating their potential as nitrogen-fixing microorganisms (Fig. 3). These included *Cyanobacteria* (*n* = 4), *Actinomycetota* (*n* = 1), *Gammaproteobacteria* (*n* = 2), and *Desulfobacterota* (*n* = 6). All of these MAGs, except for bin.276, were confirmed to be free of chimerism based on GUNC analysis (Data S4). Most of these MAGs (9 out of 13) encoded all three genes (*nifHDK*) necessary for the catalytic proteins of nitrogenase, with exceptions being bin.133, bin.237, bin.249, and bin.70. Moreover, 10 of the MAGs also possessed pathways for CO_2_ fixation, suggesting their dual role in both N_2_ and CO_2_ fixation. Specifically, four MAGs from *Cyanobacteria* and one from *Gammaproteobacteria* were linked to the Calvin cycle, while five *Desulfobacterota* MAGs encoded the Wood–Ljungdahl pathway. These results indicate that the diazotrophs represented by these MAGs likely possess autotrophic capabilities, contributing to the biogeochemical cycling of both N and C within seagrass sediments. Their ability to fix C may provide these microorganisms with a competitive advantage in their ecological niche, ultimately enhancing overall productivity and nutrient availability in these ecosystems.

**Fig 4:**
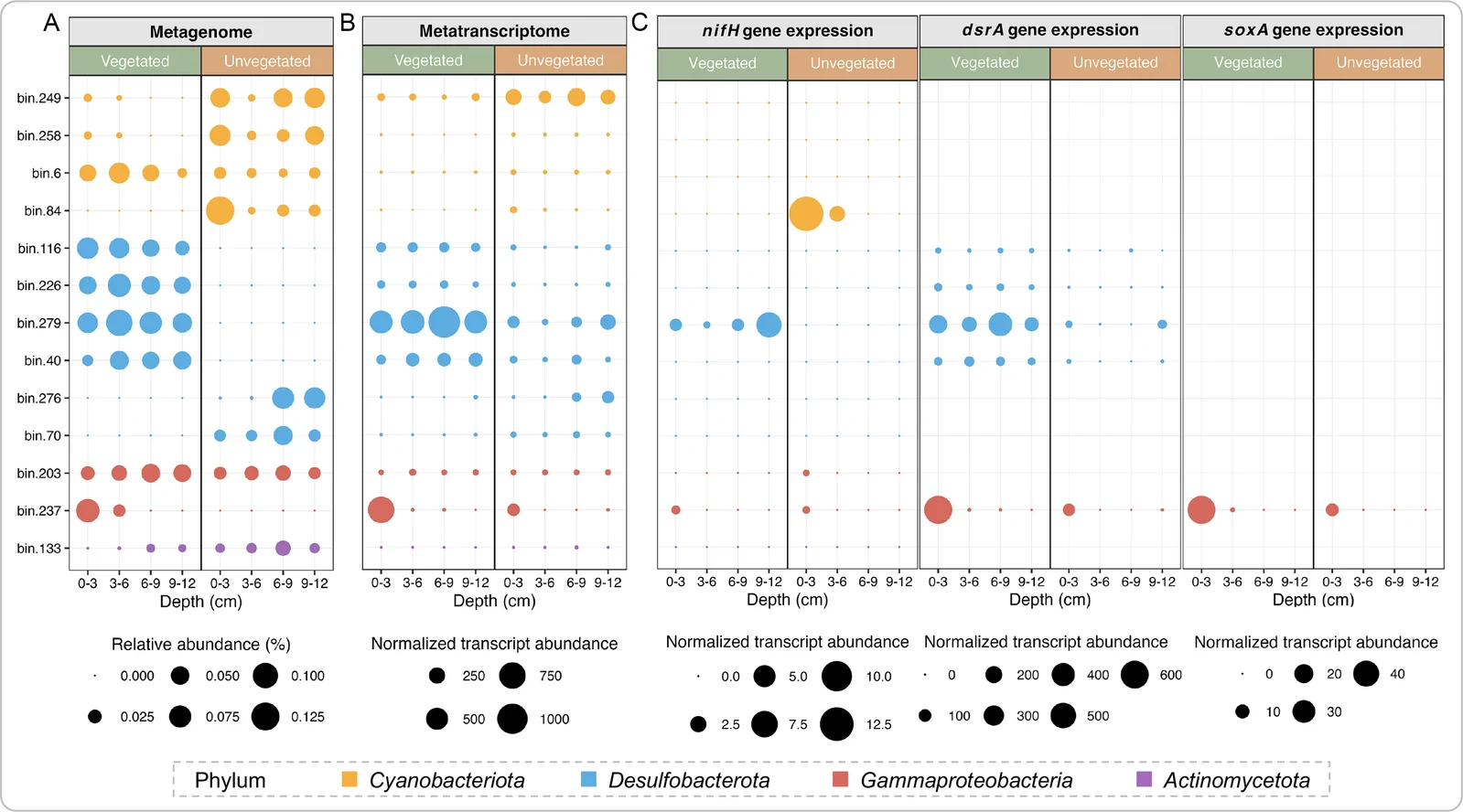
Relative abundance, transcript expression, and transcript abundance of genes for 13 diazotroph MAGs at varying depths in seagrass-vegetated and unvegetated sediments. (**A**) Relative abundance of each MAG. (**B**) Transcript expression levels for each MAG. (**C**) Transcript abundance of genes involved in nitrogen fixation and sulfur metabolism, including the nitrogenase gene (*nifH*), the dissimilatory sulfite reduction gene (*dsrA*), and the thiosulfate oxidation gene (*soxA*).

An analysis of the distribution of the 13 diazotrophs in vegetated and unvegetated sediments revealed their widespread presence across various depths (Fig. 4A and Data S6). Notably, diazotrophic sulfate-reducing *Desulfobacterota* was found to be abundant at all depths within vegetated sediments, suggesting its significant role in N_2_ fixation in these environments. Similarly, diazotrophic sulfur-oxidizing *Gammaproteobacteria* displayed high abundance throughout all sediment depths in vegetated areas, especially in the surface layers. This notable abundance indicates that *Gammaproteobacteria* may play a crucial role in N_2_ fixation within the upper layers of vegetated sediments.

To further evaluate the activity of these diazotrophs, we analyzed metatranscriptomes from various depths in both vegetated and unvegetated sediments, mapping them to nitrogenase-encoding MAGs (Fig. 4B and Data S7). Our analysis revealed high expression levels of diazotrophic sulfur-oxidizing *Gammaproteobacteria* in the surface layers of vegetated sediments. In contrast, diazotrophic sulfate-reducing *Desulfobacterota* demonstrated significant expression across all depths in vegetated sediments, with particularly elevated levels observed in the deeper layers. These findings suggested that both sulfur-oxidizing *Gammaproteobacteria* and sulfate-reducing *Desulfobacterota* are likely active nitrogen-fixing microorganisms in seagrass sediments.

Moreover, we assessed the *in situ* gene expression of these diazotrophs, revealing that certain diazotrophs co-transcribed the *nifH* gene alongside S metabolism genes (either sulfite reduction or sulfide/thiosulfate oxidation) in vegetated sediments (Fig. 4C and Data S8). Specifically, *Desulfobacterota* (bin.279) consistently transcribed the *nifH* gene and the sulfite reduction gene (*dsrA*) across all depths of the vegetated sediments, with a slight increase in transcript levels noted in the deeper layers. In contrast, *Gammaproteobacteria* (bin.237) showed high transcript levels of the *nifH* gene, along with genes related to sulfide oxidation (*dsrA*) and thiosulfate oxidation (*soxA*) in the surface layers of the vegetated sediments. In the deeper layers, however, very few or no transcripts of these genes were detected. These variations in gene expression suggest distinct functional roles that these sulfur-cycling diazotrophs play in N and S cycling across seagrass sediments. Additionally, all *Desulfobacterota* detected in vegetated sediments (bin.116, bin.226, bin.279, and bin.40) expressed genes related to the Wood–Ljungdahl pathway throughout all vegetated sediment depths, with bin.279 showing higher transcript levels. Conversely, only minimal transcript levels of the *rbcL* gene (a marker for the Calvin cycle) were detected in a *Gammaproteobacteria* MAG (bin.203) in vegetated sediments. Collectively, our findings indicate that sulfur-cycling microorganisms may dominate diazotroph communities in these ecosystems, actively expressing genes associated with both N and S metabolic pathways.

### Metabolic potential of sulfur-cycling diazotrophs

**Fig 5:**
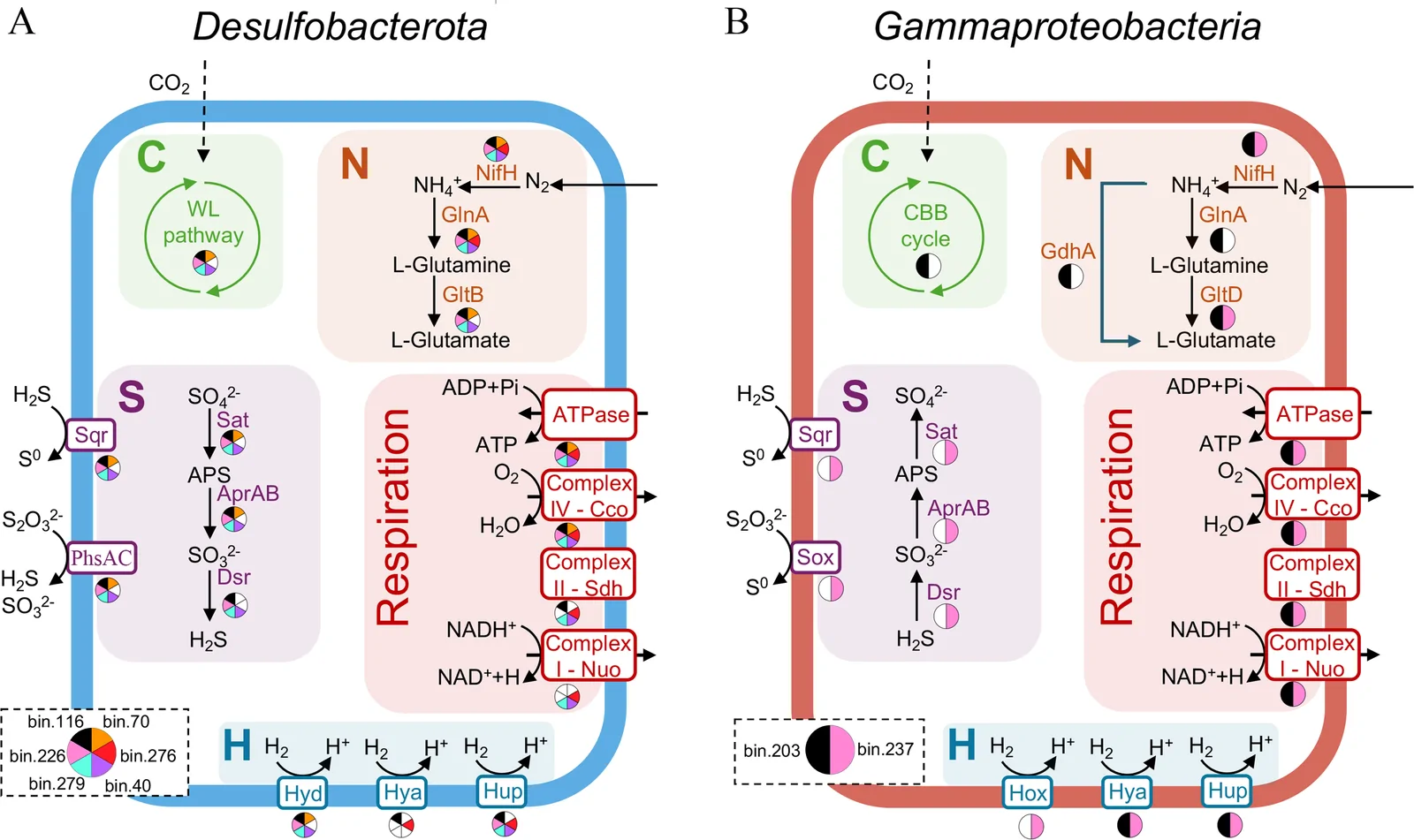
Reconstructions of the metabolic capacities for sulfur-cycling diazotroph MAGs. Metabolic capacities for *Desulfobacterota* (**A**) and *Gammaproteobacteria* (**B**) are illustrated. Circular diagrams indicate the presence (colored) or absence (white) of the corresponding genes in each MAG.

We conducted genomic analyses of MAGs from sulfur-cycling diazotrophs to explore their metabolic capabilities (Fig. 5 and Data S9). Our results indicate that sulfate-reducing *Desulfobacterota* could utilize hydrogen and sulfide as electron donors, while employing oxygen, thiosulfate, and sulfate as terminal electron acceptors to produce ATP for N_2_ fixation in vegetated sediments (Fig. 5A). The *Desulfobacterota* MAGs detected in vegetated sediments, specifically bin.116, bin.226, bin.279, and bin.40, contained the gene that encodes sulfide:quinone oxidoreductase (Sqr), an enzyme crucial for the oxidation of sulfide to elemental S (65). Additionally, these MAGs included both Group 1 [NiFe]-hydrogenase (Hyd) and Group 2 [NiFe]-hydrogenase (Hup), and bin.116 also included an additional Group 1 [NiFe]-hydrogenase (Hya), indicating their genetic potential to use hydrogen as an energy source (66). In terms of electron acceptors, these MAGs were equipped with genes for cbb3-type cytochrome oxidases (CcoNO), with bin.279 also exhibiting genes for caa3-type cytochrome c oxidases (CoxAB). The cbb3-type oxidase functions as a high-affinity terminal oxygen reductase, effective under microoxic to anoxic environments, in contrast to the low-oxygen-affinity caa3-type oxidase, which is typically activated under oxic environments (67, 68). All of these MAGs also contained genes for cytochrome bd ubiquinol oxidase (CydAB), enabling growth in microoxic environments due to its high oxygen affinity (69). Moreover, the presence of genes associated with dissimilatory sulfate reduction suggests that these organisms are genetically equipped to reduce sulfate (70), and the identification of genes for thiosulfate reductase (PhsAC) indicates their capability to reduce thiosulfate as well (71).

In contrast, sulfur-oxidizing *Gammaproteobacteria* demonstrated their ability to generate ATP for N_2_ fixation by using sulfide, thiosulfate, and hydrogen as electron donors, while employing oxygen as terminal electron acceptors (Fig. 5B). For instance, bin.237 possessed the *sqr* gene and genes for dissimilatory sulfite reductase (oxidative *dsrAB* type), indicating an ability to oxidize sulfide (72). This MAG also encoded several subunits of the Sox system (SoxAZY), suggesting its genetic capacity to oxidize thiosulfate (73). Both *Gammaproteobacteria* MAGs, namely bin.203 and bin.237, were found to harbor Group 1 [NiFe]-hydrogenase (Hya) and Group 2 [NiFe]-hydrogenase (Hup). Regarding electron acceptors, both MAGs featured genes encoding cbb3-type cytochrome oxidases (CcoNO).

### Implications of sulfur-cycling diazotrophy in seagrass sediments

Our study offers new insights into N_2_ fixation in seagrass sediments, emphasizing the roles of both sulfate-reducing and sulfur-oxidizing bacteria. Traditionally, N_2_ fixation in seagrass ecosystems has been primarily linked to heterotrophy, and especially with sulfate reduction (15–18). Previous research utilizing DNA and RNA amplicons has proposed sulfate-reducing bacteria as the primary N_2_ fixers in seagrass sediments (21, 22). In this study, we demonstrate that sulfur-oxidizing bacteria may also constitute a significant portion of the diazotroph community within seagrass sediments. Additionally, the diazotrophic sulfur-oxidizing *Gammaproteobacteria* in these sediments transcribed the nitrogenase gene *nifH*, alongside the genes for sulfide oxidation (*dsrA*) and thiosulfate oxidation (*soxA*). To the best of our knowledge, our study is among the first to use a multi-omics approach to reconstruct genomes and evaluate gene expression of uncultivated diazotrophic sulfur-cycling bacteria in seagrass sediments. We describe MAGs from diazotrophic sulfate-reducing and sulfur-oxidizing bacteria that actively express genes involved in both N_2_ fixation and dissimilatory S processes. Thus, we proposed that both sulfate-reducing and sulfur-oxidizing bacteria contribute to N_2_ fixation in seagrass sediments, expanding our understanding of the microbial dynamics in these vital ecosystems.

We complied and curated publicly available metagenomic data from seagrass sediments, noting that only 39 metagenomes from seven globally distributed seagrass regions have been documented to date (Data S1). Our analysis revealed MAGs harboring the *nifH* gene, along with those related to sulfide and thiosulfate oxidation, in the sediments of *Z. marina* and *P. sinuosa* (74–76). This supports our findings regarding the potential role of sulfur-oxidizing bacteria in N_2_ fixation within seagrass sediments. However, none of these MAGs contained both the *nifH* gene and genes for sulfate reduction. Notably, only four MAGs were retrieved from the sediments of *P. sinuosa* (74). In studies that included only one or two metagenomic samples, fewer than 10 MAGs were recovered, and none encoded the *nifH* gene. Consistent with our findings, MAGs containing both sulfur-cycling genes (for either thiosulfate oxidation or sulfate reduction) and nitrogenase genes have been previously reported in *Z. japonica*-colonized and adjacent unvegetated sediments, yet their distribution, function, and gene expression remain unclear (24). Given the limited metagenomic and metatranscriptomic data available, further research is essential to better understand the diazotrophic populations in these vital ecosystems.

The presence of seagrass appears to promote the dominance of sulfur-cycling bacteria within diazotroph communities. Both sulfate-reducing and sulfur-oxidizing bacteria are prevalent and transcriptionally active in diazotroph communities of vegetated sediments. In contrast, unvegetated sediments are primarily dominated by *Cyanobacteria*, with *Desulfobacterota* also comprising a significant portion of the diazotroph communities. Comparing biogeochemical characteristics between these two environments, we found that the vegetated sediments exhibited an increased C:N ratio, along with higher levels of organic C and NH_4_^+^ compared to unvegetated sediments. Particularly, in subsurface layers where SO_4_^2−^ concentrations were lower in vegetated sediments, significantly higher N_2_ fixation rates were detected. This suggests a link between N_2_ fixation and S metabolism, specifically in vegetated sediments. These findings underscore the critical role of seagrass in shaping microbial diversity and metabolic interactions, thereby influencing biogeochemical cycling in coastal ecosystems.

Moreover, our results suggest a coupling between N_2_ fixation and the processes of sulfate reduction and S oxidation in seagrass sediments, highlighting the interconnectedness of these metabolic pathways. Additionally, research using 16S rRNA gene amplicon sequencing and metatranscriptomics has shown that nitrogenase genes (*nifHDK*) are expressed by sulfur-oxidizing *Gammaproteobacteria*, specifically *Arcobacter* sp., in the roots and leaves of *Z. marina* and *Z. japonica* (77). Notably, similar couplings between N_2_ fixation and S metabolism (both oxidation and reduction) have also been reported in other habitats, such as the roots of the salt marsh foundation plant *Spartina alterniflora* (56), suggesting a broader ecological significance.

**Fig 6:**
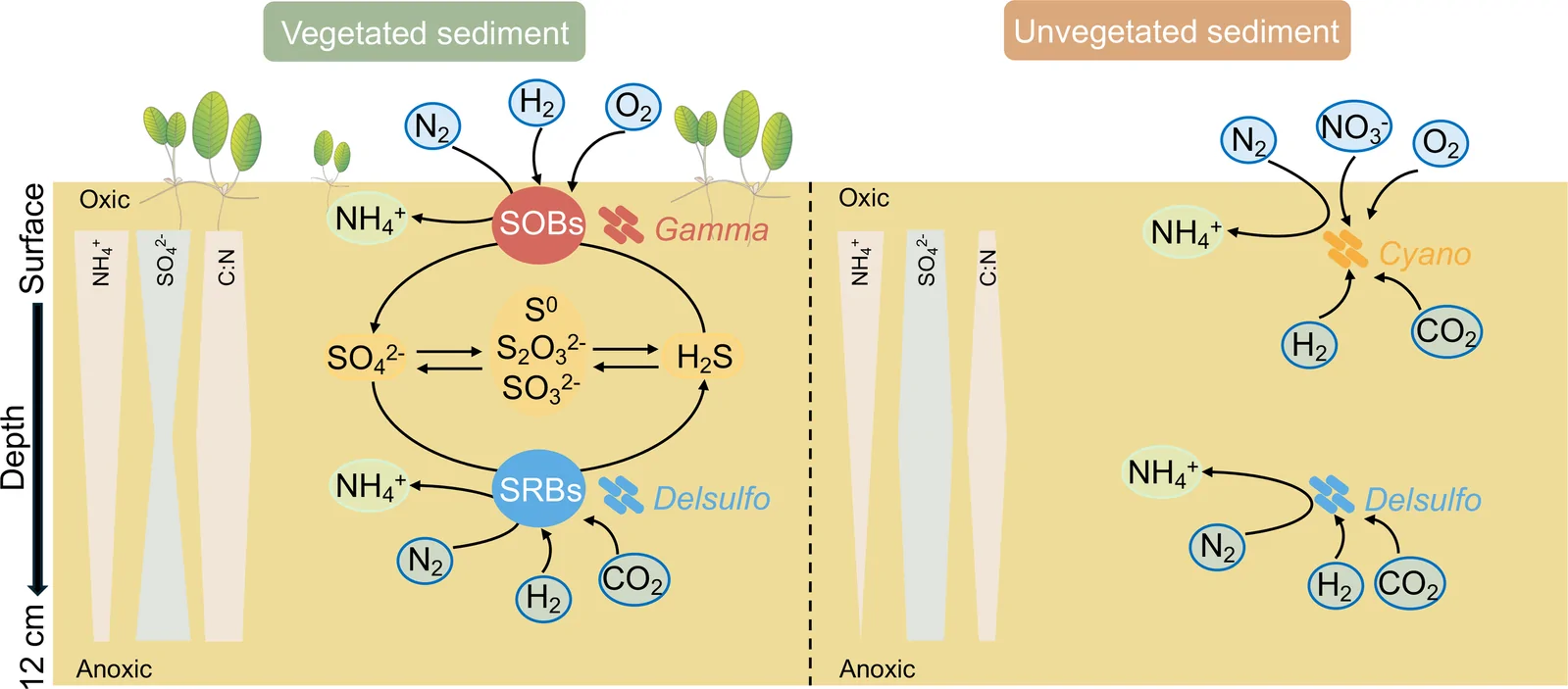
Schematic diagram illustrating depth-related microbial nitrogen fixation in sediments dominated by the seagrass *Halophila ovalis* and adjacent unvegetated sediments. In vegetated sediments, sulfur-cycling bacteria are key components of diazotroph communities, with diazotrophic sulfur-oxidizing *Gammaproteobacteria* (*Gamma*) predominant in the surface layers and sulfate-reducing *Desulfobacterota (Desulfo)* thriving in deeper layers, both of which play critical roles in nitrogen fixation. Conversely, unvegetated sediments are primarily dominated by *Cyanobacteria* (*Cyano*) and *Desulfobacterota*. In these environments, the nitrogen fixation activities of these organisms do not seem to be associated with S metabolism, which represents a notable distinction from the dynamics observed in vegetated sediments. SOBs, sulfur-oxidizing bacteria; SRBs, sulfate-reducing bacteria.

To illustrate the dynamics of N_2_ fixation in seagrass sediments, we propose a conceptual model of depth-related microbial N_2_ fixation (Fig. 6). Previous studies have demonstrated that oxygen depletion in seagrass sediments occurs rapidly, typically at depths ranging from 2 to 5 mm (6, 78, 79). The resulting anoxic sediment conditions create an ideal environment for diazotrophic sulfate-reducing bacteria in deeper layers of vegetated sediments. In these layers, sulfate-reducing *Desulfobacterota* are likely to dominate the diazotroph communities. They could utilize hydrogen and sulfide as electron donors while employing oxygen, sulfate, and thiosulfate as terminal electron acceptors. This metabolic activity may lead to low SO_4_^2−^ concentrations and elevated NH_4_^+^ levels in the subsurface sediments. Additionally, these *Desulfobacterota* can genetically convert carbon dioxide into organic C. The upward diffusion of sulfide produced by sulfate-reducing bacteria may create a dynamic interaction with sulfur-oxidizing bacteria in the upper layers of vegetated sediments (80). In these surficial sediments, sulfur-oxidizing *Gammaproteobacteria* may dominate the diazotroph communities, which could use sulfide and hydrogen as electron donors and oxygen as the terminal electron acceptor. This metabolic process likely results in high concentrations of NH_4_^+^ and SO_4_^2−^ in the upper sediment layers, further illustrating the intricate interplay between these microbial communities and their contributions to the C, N, and S cycles in seagrass ecosystems. In contrast, unvegetated sediments tended to be dominated by *Cyanobacteria* and *Desulfobacterota*. In these environments*,* the N_2_ fixation activities of these organisms do not appear to be linked with S metabolism, which is a notable distinction from their vegetated counterparts (Data S9). The proposed model of microbial N_2_ fixation and its interactions within seagrass sediments highlights the importance of these communities in nutrient cycling. However, it should be further validated through direct process-level measurements and culture experiments to enhance our understanding of these intricate dynamics.

### Conclusions

By integrating biogeochemistry with a multi-omics approach, our study sheds light on biological N_2_ fixation in seagrass meadows, which are critical blue carbon ecosystems in coastal and estuarine regions. We found that N_2_ fixation rates are markedly higher in seagrass-vegetated sediments than in adjacent unvegetated sediments and surrounding waters, indicating that the seagrass sediments act as hotspots for N_2_ fixation. Our research demonstrates the distribution, metabolic potentials, and activity of diazotrophs, suggesting the ecological importance of sulfur-cycling bacteria in N metabolism within seagrass sediments. Notably, both sulfate-reducing and sulfur-oxidizing bacteria dominated the diazotroph communities and actively expressed genes related to N_2_ fixation and S metabolism in these environments. These microbes may play a crucial role in the biogeochemical cycling of C, N, and S by providing bioavailable N in seagrass sediments that are rich in C and S. To further validate the roles of these diazotrophs, isolating and cultivating the dominant N_2_-fixing microorganisms from seagrass sediments will be essential. Additionally, future research should aim to quantify the contributions of sulfur-cycling diazotrophs to the overall N_2_ fixation *in situ*.

## Data Availability

The metagenomic and metatranscriptomic data used in this study have been deposited in the National Center for Biotechnology Information (NCBI) under BioProject no. PRJNA1344282 and PRJNA1344283, respectively. All MAGs have been deposited in figshare (https://doi.org/10.6084/m9.figshare.30329509.v1). All other data supporting the findings of this study are available within the article and its supplemental material.

## SUPPLEMENTAL MATERIAL

Data S1 aem.00993-26-s0001.xlsx Metagenomic data analyzed in this study, including NCBI accession number, species, latitude, longitude, and source.

Data S2 aem.00993-26-s0002.xlsx Vertical profiles of various environmental characteristics in seagrass-vegetated and unvegetated sediments.

Data S3 aem.00993-26-s0003.xlsx Summary of 107 *nifH* genes from assembled contigs obtained in current study.

Data S4 aem.00993-26-s0004.xlsx Genomic statistics of 305 MAGs.

Data S5 aem.00993-26-s0005.xlsx Copy number of selected genes in the carbon, nitrogen, and sulfur cycles from all MAGs.

Data S6 aem.00993-26-s0006.xlsx Relative abundance for 13 diazotroph MAGs at varying depths in seagrass-vegetated and unvegetated sediments.

Data S7 aem.00993-26-s0007.xlsx Normalized transcript abundance for 13 diazotroph MAGs at varying depths in seagrass-vegetated and unvegetated sediments.

Data S8 aem.00993-26-s0008.xlsx Normalized transcript abundance of genes in nitrogen fixation, sulfur reduction/oxidation, and carbon fixation for 13 diazotroph MAGs at varying depths in seagrass-vegetated and unvegetated sediments.

Data S9 aem.00993-26-s0009.xlsx Copy number of genes in diazotroph MAGs from Desulfobacterota, Gammaproteobacteria, and Cyanobacteria.

Supplemental figures aem.00993-26-s0010.docx Fig. S1 to S4.

ASM does not own the copyrights to Supplemental Material that may be linked to, or accessed through, an article. The authors have granted ASM a non-exclusive, world-wide license to publish the Supplemental Material files. Please contact the corresponding author directly for reuse.
