# Supplementary material for: Sulfur-cycling diazotrophs dominate nitrogen fixation in seagrass sediments
Source: Appl Environ Microbiol. 2026 Jul 15;92(8):e00993-26. doi: 10.1128/aem.00993-26 (PMC13488301; doi:10.1128/aem.00993-26)
Supplement: Supplementary file 1 [file aem.00993-26-s0010.docx]

**Supporting Information for**

**Sulfur-cycling diazotrophs dominate nitrogen fixation in seagrass sediments**

Lixia Deng, Zhicheng Ju, Jiawei Chen, Yuxuan Lin, Weiguo Zhou, Sangwook Scott Lee, Charmaine C. M. Yung, Hongbin Liu*

Corresponding author: Dr. Hongbin Liu, [liuhb@ust.hk](mailto:liuhb@ust.hk)

**Supplementary Figures**

**Figure S1** (A) A district map of Hong Kong showing the sampling location of the studied *Halophila ovalis* seagrass meadow. (B) A view of the seagrass meadow and the surrounding mangroves.

**Figure S2 Vertical profiles of various environmental characteristics in seagrass-vegetated and unvegetated sediments**. The concentrations of TOC (A) and TN (B), the C:N ratio (C), and the concentrations of NH_4_^+^ (D), NO_3_^-^+NO_2_^-^ (E), and SO_4_^2-^ (F) across different depths are shown.

**Figure S3 Nitrogen fixation rates in surrounding seawater, unvegetated sediments, and vegetated sediments, shown as acetylene reduction (nmol N_2_ g^-1^ d^-1^).** In boxplots, the boxes are defined by the upper and lower quartiles, and the median is shown by a horizontal line within the box. The whiskers reach the most extreme data points, which are within 1.5 times the interquartile range. Different letters represent statistical significance between groups based on pairwise Mann-Whitney tests at *p*<0.05.

**Figure S4 Phylogenetic tree of the *dsrAB* gene retrieved from MAGs.** Genes were aligned to a reference database, and the phylogenetic tree was constructed using IQ-TREE v1.6.12. Phylogenetic placement within the tree was used to classify the *dsrAB* gene into oxidative and reductive types.

**Supplementary Data**

Supplementary Data 1. Metagenomic data analyzed in this study, including NCBI accession number, species, latitude, longitude, and source

Supplementary Data 2. Vertical profiles of various environmental characteristics in seagrass-vegetated and unvegetated sediments

Supplementary Data 3. Summary of 107 *nifH* genes from assembled contigs obtained in current study

Supplementary Data 4. Genomic statistics of 305 MAGs

Supplementary Data 5. Copy number of selected genes in the carbon, nitrogen, and sulfur cycles from all MAGs

Supplementary Data 6. Relative abundance for 13 diazotroph MAGs at varying depths in seagrass-vegetated and unvegetated sediments

Supplementary Data 7. Normalized transcript abundance for 13 diazotroph MAGs at varying depths in seagrass-vegetated and unvegetated sediments

Supplementary Data 8. Normalized transcript abundance of genes in nitrogen fixation, sulfur reduction/oxidation, and carbon fixation for 13 diazotroph MAGs at varying depths in seagrass-vegetated and unvegetated sediments

Supplementary Data 9. Copy number of genes in diazotroph MAGs from *Desulfobacterota*, *Gammaproteobacteria*, and *Cyanobacteria*
